# Supplementary material for: Transcriptome Analysis of Leg Muscles and the Effects of ALOX5 on Proliferation and Differentiation of Myoblasts in Haiyang Yellow Chickens
Source: Genes (Basel). 2023 Jun 1;14(6):1213. doi: 10.3390/genes14061213 (PMC10298623; doi:10.3390/genes14061213)
Supplement: Supplementary file 1 [file genes-14-01213-s001.zip › genes-2397482-supplementary.pdf]

Supplementary files

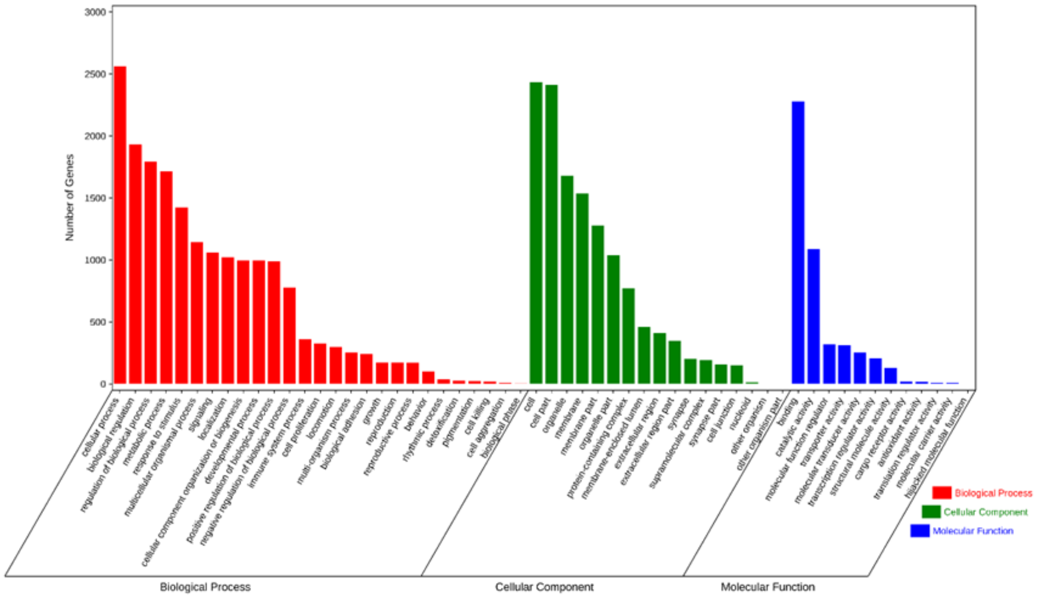

Figure S1. Gene Ontology enrichment analysis of all the differentially expressed genes in male chickens.

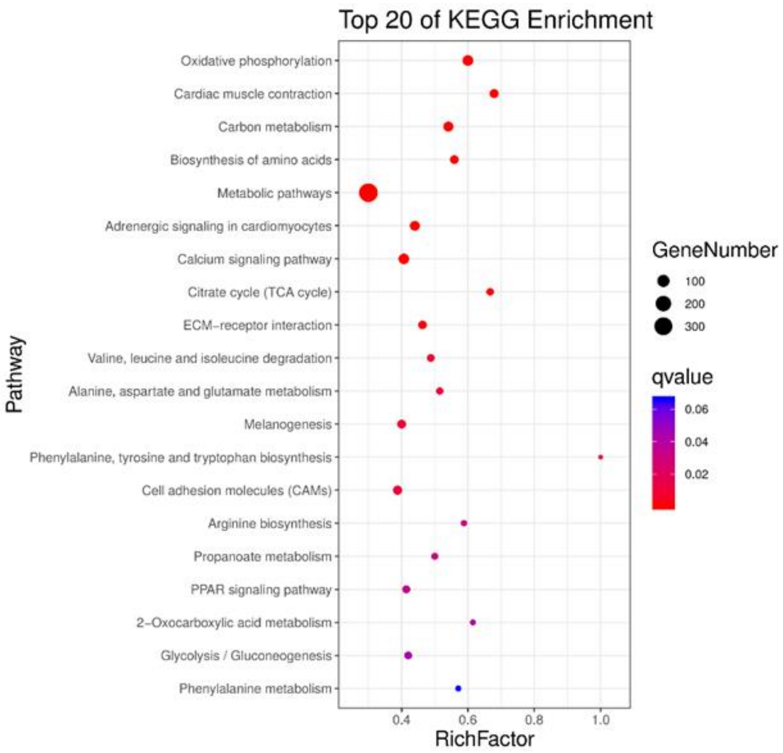

Figure S2. The top 20 enriched pathways of DEGs in male chicken.

The enrichment analysis of KEGG pathway of the top 20 differential genes. Rich Factor, the higher the value, the greater the enrichment degree. The color depth represents the P-value, and the darker the color, the more significant the difference. The dot size represents the number of genes enriched in the pathway.

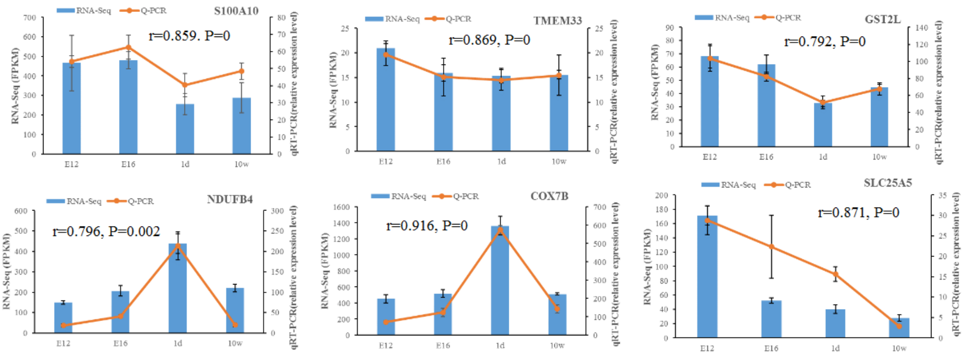

Figure S3. The validation of differentially expressed genes through quantitative reverse-transcription-PCR.

R represents correlation, P represents value judgment. |r| value, the greater the correlation, the better. Present positive correlation between positive said, negative correlation between negative said. The smaller the P value, the more significant the difference.

Table S1. Primer Sequences for quantitative reverse-transcription PCR

Gene name	Primer sequence (5′-3′)	Product (bp)	Annealing (°C)
S100A10	TTCCACAAATACGCGGGTGA<br>AGCCACCAGTGAGAAGAAGC	186	60
COX7B	TATGGAAATCTGGTGCTGATCG<br>CAGTTCATTGCAAGCAAAGGC	155	60
SLC25A5	TACCAGGGCTTCAACGTGTC<br>CCGAACGGTATCGAAGGGAT	186	60
NDUFB4	AACCGCTACGTTTCGTTGCC<br>CGGAAAGTGGGGTACACGTT	218	60
GST2L	TGCTGGAGAACCAGGTGATG<br>AACCACTTTCTGTCCCCCAG	148	60
TMEM33	GACACAGCAATGTGGATTTCCC<br>CTTTGGTGTAGTCGGAGAGCA	149	60
β-actin	CAGCCATCTTTCTTGGGTAT<br>CTGTGATCTCCTTCTGCATCC	169	60
HSP70	TCTGCTCCTGTTGGATGTC<br>TGGGAATGGTGGTGTTACG	95	60
ALOX5	GCGTGGATGCCAATAAGAC<br>GAGACTAGGTGTGTCCGTAAGAG	253	60
MYOD	GCTACTACACGGAATCACCAAAT<br>CTGGGCTCCACTGTCACTCA	200	60
MEF2C	GGAGGATACCCATCAGCCAT<br>AGCCAGTCACAGAACCAAGA	124	60

MYOG	CGGAGGCTGAAGAAGGTGAA<br>CGGTCCTCTGCCTGGTCAT	320	60

Table S2. The results of RNA quality detection

Sample name	RIN	OD260/280	OD260/230	28S/18S
E12-M1	7.6	1.974	2.133	0.8
E12-M2	7.4	1.967	2.068	0.7
E12-M3	8.4	1.991	2.009	1.5
E16-M1	9.2	1.948	1.887	1.2
E16-M2	9.5	1.955	2.227	1.3
E16-M3	9.5	1.922	1.703	1.4
1d-M1	9	1.885	1.989	1.1
1d-M2	9.3	1.959	2.204	1.2
1d-M3	8.9	1.954	2.076	1.2
10W-M1	8.6	1.881	1.82	1.1
10W-M2	9.1	1.921	2.165	1.1
10W-M3	8.8	1.875	2.283	1.2

Table S3 The results of comparison with reference genome for clean reads

Sample name	Total reads	Total mapped	Multiple mapped	Uniquely mapped
E12-M1	61790372	53387806(86.4%)	22012191(3.56%)	51186515(82.84%)
E12-M2	65909504	56686510(86.01%)	2576316(3.91%)	54110194 (82.1%)
E12-M3	64494886	54579542 (84.63%)	3880488 (6.02%)	50699054 (78.61%)
E16-M1	56999228	50121611 (87.93%)	1901087 (3.34%)	48220524 (84.6%)
E16-M2	52273528	45470227 (86.99%)	1718286 (3.29%)	43751941 (83.7%)
E16-M3	55850416	48895846 (87.55%)	1934697 (3.46%)	46961149 (84.08%)
1d-M1	61428646	53389831 (86.91%)	1904079 (3.1%)	51485752 (83.81%)
1d-M2	69820058	60119337 (86.11%)	2243311 (3.21%)	57876026 (82.89%)
1d-M3	64664226	56528888 (87.42%)	1909429 (2.95%)	54619459 (84.47%)
10W-M1	63540482	54672509 (86.04%)	2112197 (3.32%)	52560312 (82.72%)
10W-M2	54365192	47684092 (87.71%)	1541352 (2.84%)	46142740 (84.88%)
10W-M3	64191268	54433294 (84.8%)	2252817 (3.51%)	52180477 (81.29%)

Note：(1) Total reads：The number of sequences filtered by sequencing data. (2) Total mapped：Statistics on the number of sequenced sequences that can be located on the genome. (3) Multiple mapped: The number of sequencing sequences with multiple alignment locations on the reference sequence. This percentage is usually less than 10%. (4) Uniquely mapped: The number of sequencing sequences with unique alignment locations on the reference sequences is counted.
